# Evaluating the Impact of Two Different Diets on the Protein Profile of the Brain, Liver, and Intestine of the Barramundi

**DOI:** 10.3390/proteomes14010006

**Published:** 2026-01-29

**Authors:** Mohadeseh Montazeri Shatouri, Igor Pirozzi, Pinar Demir Soker, Zeshan Ali, Ardeshir Amirkhani, Paul A. Haynes

**Affiliations:** 1 School of Natural Sciences, Macquarie University, North Ryde, NSW 2109, Australia; mohadeseh.montazerishatouri@hdr.mq.edu.au (M.M.S.); zeshan.ali@hdr.mq.edu.au (Z.A.); 2 ARC Training Centre for Facilitated Advancement of Australian Bioactives (FAAB), Macquarie University, North Ryde, NSW 2109, Australia; 3 Port Stephens Fisheries Institute, NSW Department of Primary Industries and Regional Development, Taylors Beach, NSW 2316, Australia; igor.pirozzi@dpird.nsw.gov.au; 4 Skretting Australia, Cambridge, TAS 7170, Australia; 5 Australian Proteome Analysis Facility, Macquarie University, North Ryde, NSW 2109, Australia; ardeshir.amirkhani@mq.edu.au

**Keywords:** proteomics, commercial feed, aquaculture, barramundi, brain, liver, intestine, diet

## Abstract

Background: Commercial feed formulations are increasingly being evaluated for their nutritional impacts on aquaculture species, yet the molecular consequences of commonly used commercial diets remain underexplored. Methods: This study investigated the effects of two commercial diets, diet A (higher land animal protein) and diet B (higher fish meal content), on the protein profile in the brain, liver, and intestine of barramundi (*Lates calcarifer*). A 12-week feeding trial was conducted with controlled water quality, and proteomic profiling was performed using data-independent acquisition. Results: Differential analysis revealed consistent changes between diets across all tissues, with a higher percentage of differentially abundant proteins observed in between-diet comparisons (12.99% in brain, 12.73% in liver, and 16.59% in intestine) than within-diet controls (<8%), confirming a measurable dietary effect size. In total, 3901 proteins in the brain, 3660 in the liver, and 5025 in the intestine were quantified. Functional enrichment highlighted upregulation of ferroptosis pathways, downregulation of apelin signaling in the brain, and increased digestive proteases in the liver. ICP-MS confirmed elevated iron concentrations in the brain, liver, and intestine of fish fed on diet B. Conclusions: These findings demonstrate that molecular pathways linked to iron metabolism, digestion, and growth regulation are very sensitive to dietary composition, highlighting how proteomics can help identify subtle impacts of compositional differences in aquaculture feeding. Although physiological parameters did not differ significantly, the proteomic alterations observed across tissues likely indicate organ-specific metabolic adaptations to the differing nutrient availability between diets.

## 1. Introduction

Barramundi (*Lates calcarifer*) (Bloch, 1790) are native to the Indo-Pacific region. They are found as far north as Taiwan, as far south as Fraser Island on the Australian east coast, eastward as far as southeastern Papua New Guinea, and westward as far as the Persian Gulf. In the wild, the fish are a diadromous species, returning to estuarine or marine water to breed [1]. They are now also an important aquaculture species in the Indo-Pacific region, produced in both fresh and saltwater. The species is grown to a harvest size of between 400 and 4000 g, depending on the production system and market [2].

To sustain growth, the barramundi, like all animals, requires the dietary intake of certain nutrients. The dietary concentration required of these nutrients is largely driven by the energetic content of the food eaten by the fish. Several studies have been undertaken to examine the requirements for protein in the barramundi diets. Most of these studies suggest a relatively high protein requirement, consistent with the carnivorous/piscivorous nature of the fish [3]. Despite these insights, research on the individual amino acid requirements remains limited; to date, only four of the ten essential amino acids have been thoroughly investigated, with existing estimates focusing on methionine/total sulfur amino acids, lysine, arginine, and tryptophan [4]. In addition, lipids—being the most energy-dense nutrient—have received considerable attention, as they provide nearly double the energy of protein and significantly more than carbohydrates [2].

Proteomics has been used to investigate food intake and appetite in other monogastric animals [5]. Omic-based studies on the barramundi have so far been limited to phenotype characterization [6], speciation and adaptation [7], sensory and volatile characteristics [8], and hepatic transcriptomes [9]. In a previous study, the use of animal protein sources as a replacement for fishmeal in fish diets had a positive impact on the feed conversion ratio, variable growth rate, final weight, and survival rate of different types of fish species of different size groups [10]. However, little is known about how changes in physiological processes associated with food intake in fish are reflected at the proteome level. Advances in proteomic technologies now offer a powerful means to explore the molecular mechanisms underpinning these nutritional requirements. High-throughput proteomics allows for the simultaneous profiling of thousands of proteins, providing insights into the regulatory networks and metabolic pathways that are modulated by dietary composition [11]. This type of molecular-level investigation is critical because it can reveal early biomarkers of nutritional stress or suboptimal dietary formulations well before changes in growth performance or health become apparent.

In this study, we conducted a 12-week feeding trial using two different commercial diets (designated diet A and diet B) provided by Skretting Australia. These diets differed in ingredient composition but shared the same crude protein and crude fat levels (crude protein/crude fat: 45/20). Diet A contained less fish meal and more land-animal protein sources than diet B and was also characterized by lower levels of methionine as an essential amino acid. These compositional differences provided an opportunity to investigate how subtle shifts in dietary protein quality and ingredient origin affect tissue-level protein abundance and nutrient regulation.

Our aim was to compare the proteomic profiles of key tissues, including brain, liver, and intestine, to gain a more comprehensive understanding of how diet influences metabolic regulation and cellular function in the barramundi. These three tissues were selected for their pivotal roles in maintaining overall physiological balance [12]. The liver plays a central role in the digestion, transport, metabolism, and storage of nutrients, as well as detoxification, immunity, and health [13]. The brain integrates neural and hormonal signals to regulate feeding behavior [14], and the intestine is the primary site for nutrient digestion, motility, secretion, and absorption [12].

We hypothesized that the nutrient composition differences between diet A and diet B would lead to distinct alterations in proteomic profiles of different tissues, and this study was designed to assess the nature and extent of those changes. The insights gained from this study will improve our understanding of the dietary requirements of farm-raised barramundi.

## 2. Materials and Methods

### 2.1. Feeding, Handling, and Treatment of Barramundi

The feeding trial was conducted over a period of 12 weeks, using two different commercial diets manufactured by Skretting Australia (Cambridge, TAS, Australia). The relative compositional differences between the diets are detailed in Table 1.

Levels of other components were the same between the two diets, including crude protein, crude fat, lupin, faba, total amino acids, total fatty acids, starch, fiber, vitamins, zinc, copper, and antioxidants.

The experimental design involved stocking 25 barramundi, each weighing approximately 500 g, into two 1000 L aquaculture tanks per diet treatment. The experimental tanks were integrated within a 30,000 L recirculating aquaculture system, which provided mechanical and biological filtration and allowed control of water flow. All tanks of fish were hand-fed twice daily during weekdays (9:00 am and 2:00 pm) and once daily on weekends (8:30 am). Feeding was conducted to apparent satiation over approximately one hour and determined when pellets began to accumulate uneaten on the surface water. The remaining uneaten pellets were netted from tanks, counted, and feed intake adjusted using a correction factor based on average pellet weight (*n* = 200) determined for each diet.

This study was performed under the NSW DPI Fisheries Animal Care & Ethics Authority, known as ACEC-0397 Aquaculture Nutrition. Care, husbandry, and termination of fish were performed according to methods outlined in ‘A Guide to Acceptable Procedures and Practices for Aquaculture and Fisheries Research, 4th Edition, 2015’. All fish were sedated prior to handling using AQUI-S^®^ (AQUI-S New Zealand Ltd., Lower Hutt, New Zealand) and euthanized with an overdose of benzocaine solution (≥100 mg ethyl-p-amino benzoate L-1). Tissue samples from the brain, liver, and intestine of 8 fish fed on diet A and 8 fish fed on diet B were collected after the measurement of growth rate parameters. The tissue samples were randomly assigned into two groups of four from each diet to facilitate subsequent analysis using four replicates each for both within-diet and between-diet quantitative comparisons. The samples were stored on dry ice and transferred to −80 °C for storage until further processing.

### 2.2. Water Quality

Water quality variables were recorded daily (at 8:30 am and 3:30 pm) using portable electronic instruments. The average water temperature during the experiment was approximately 28 ± 1 °C. Salinity ranged from 30‰ to 33‰. Dissolved oxygen concentration remained above 7 mg L^−1^. Throughout the experiment oxygen saturation remained between 120% and 180%, and pH ranged from 6.9 to 8.4. Total ammonia nitrogen (TAN) concentration was measured regularly using a colorimetric test kit, with the average TAN concentration ≤ 0.8 mg L^−1^.

### 2.3. Calculated Production Indices

Performance indices, including growth rate, food intake, and food conversion ratios, were calculated using the formula below [15]:

Specific growth rate (% d^−1^) = (Ln (final weight) − Ln (initial weight))/days × 100;

Relative food intake (g kgBW^−1^ d^−1^) = daily food intake per fish (g)/(geometric mean body weight of fish (kg));

Food conversion ratio (FCR) = food intake per tank (g)/wet weight gain per tank (g).

The effect of dietary treatment on the performance of the barramundi was analyzed using one-way ANOVA, considering diet as a fixed factor. The level of probability for which ANOVA was considered significant was α = 0.05. Differences among significant treatment means were determined using Tukey’s Honest Significance Difference post hoc test (α = 0.05).

### 2.4. Protein Extraction from Brain, Liver, and Intestine

The tissues collected from fish grown on both diets, stored at −80 °C, were ground on dry ice. Approximately 20 mg aliquots from brain, liver, and intestine were transferred to 2 mL Eppendorf tubes kept on dry ice (8 biological replicates per tissue). A volume of 200 µL of RIPA buffer (50 mM Tris, pH 7.5, 150 mM NaCl, 1% NP40, 1 mM EDTA, 0.1% SDS containing 1% *v*/*v* protease inhibitor cocktail) was added to the tube, and the tissue was homogenized using a TissueLyser (T 10 basic ULTRA-TURRAX, IKA, Staufen, Germany for 30 s). Subsequently, the sample was sonicated twice using a probe sonicator (20 kHz, 40% amplitude, 2 × 15 s pulses, with 30-s intervals on ice). The samples were then centrifuged for 15 min at 13,000 rpm at 4 °C to remove debris. The supernatant was transferred to a clean tube. Proteins were reduced using 20 mM dithiothreitol (DTT) for 45 min. The reduced protein extract was then alkylated with 50 mM iodoacetamide for 30 min in the dark. Alkylation was stopped by adding 40 mM DTT.

Protein extracts were precipitated using methanol/chloroform. First, 800 µL of methanol (4 times the volume of the lysis buffer), 200 µL of chloroform (equal to the volume of the lysis buffer), and 600 µL of water (3 times the volume of the lysis buffer) were added to the supernatant. The mixture was vortexed and centrifuged for 1.5 min at 13,000 rpm. The upper phase was removed, and 800 µL of methanol was added to the tube, followed by centrifugation for 5 min at 13,000 rpm. The supernatant was removed, and the pellet was air-dried in a fume hood. The protein pellet was solubilized in 8 M urea.

### 2.5. Protein Quantification and Digestion

The bicinchoninic acid (BCA) assay kit (Thermo Scientific, San Jose, CA, USA) was used to measure the concentration of protein in the sample extract. From each sample, 100 µg of protein was transferred to a 1.5 mL tube. Trypsin (1 µg) was added to each sample, and digestion occurred overnight at 37 °C. Digestion was halted by adding TFA to adjust the pH to 3. Samples were desalted using SDB-RPS (3M Empore, Sigma-Aldrich, St. Louis, MO, USA) 200 µL stage tips [16]. The membranes were first conditioned with 100 µL of acetonitrile. Subsequently, 100 µL of 1% trifluoroacetic acid (TFA)/30% methanol and 100 µL of 0.2% TFA were sequentially loaded onto the membrane. Samples were loaded onto the conditioned tips and centrifuged. The columns were then washed with 0.2% TFA to remove unbound material. Tryptic peptides were eluted with 200 µL of 80% ACN and 5% ammonium hydroxide into a new tube. The eluted peptides were dried using a vacuum centrifuge and resuspended in 35 µL of 0.1% formic acid. A micro-BCA assay was performed to determine the peptide concentration.

### 2.6. Peptide Fractionation for Spectral Library Generation

A pooled sample containing 200 µg of protein was prepared for each tissue separately by combining 12.5 µg each from all 16 replicates and fractionated using high-pH RP-HPLC, with 17 fractions collected. The fractionation was performed using an Agilent 1260 HPLC system equipped with a quaternary pump (G1311B), an autosampler (G1329B) with a 900 µL injection loop and syringe, a column compartment (G1316A), a fraction collector (G1364C), and a thermostat for the autosampler and fraction collector (G1330B). The analysis was conducted using ChemStation chromatography software (version LTS 01.09). A Zorbax 300Extend-C18 column (2.1 × 150 mm, 3.5 µm, 300 Å) was used, along with a 96-well, 2 mL deep, well-rounded bottom polypropylene collection plate. The gradient elution method consisted of an 85-min gradient, starting with 97% mobile phase A (milli-Q water with 6 mM ammonium hydroxide) and 3% mobile phase B (6 mM ammonium hydroxide in 90:10 ACN: MilliQ water) for 10 min, followed by 55 min of 70% mobile phase A and 30% mobile phase B, 10 min of 30% mobile phase A and 70% mobile phase B, 5 min of 10% mobile phase A and 90% mobile phase B, and finally, 5 min of 97% mobile phase A and 3% mobile phase B, all at a flow rate of 0.3 mL/min and a column temperature of 25 °C. UV detection was performed at wavelengths of 210 nm and 280 nm.

### 2.7. Nanoflow Liquid Chromatography–Tandem Mass Spectrometry (nanoLC-MS/MS) Analysis of Pooled Samples for Library Generation

Fractions prepared by HpH reverse phase fractionation for library generation were analyzed on a Q-Exactive HF-X mass spectrometer (Thermo Fisher Scientific, San Jose, CA, USA) interfaced with an UltiMate 3000 UHPLC (Thermo Fisher Scientific) column using data-dependent acquisition (DDA) mode with a full scan followed by fragmentation of the top 10 most abundant ions. A 90-min gradient was employed, beginning with 98% mobile phase A and 2% mobile phase B for 10 min, followed by 60 min of 65% mobile phase A and 35% mobile phase B, 8 min of 5% mobile phase A and 95% mobile phase B, 5 min of 5% mobile phase A and 95% mobile phase B, and 4 min of 98% mobile phase A and 2% mobile phase B. The flow rate was set at 300 nL/min, using a 75 µm × 30 cm C18 column (ReproSil-Pur 120 C18-AQ, Dr. Maisch, Ammerbuch-Entringen, Germany) maintained at 45 °C. The analysis was conducted in positive ion mode with automatic gain control (AGC) set at 3 × 10^6^ for ion accumulation, a maximum trapping time of 50 milliseconds, a scan range of 350 to 1650 *m*/*z*, a full scan resolution of 60,000, an MS2 resolution of 15,000, higher energy collision dissociation (HCD) fragmentation of the top 10 ions, a normalized collision energy of 27.5, and dynamic exclusion enabled for 15 s.

### 2.8. nanoLC-MS/MS Data-Independent Acquisition Proteomic Analysis of Tissue Samples

Tryptic peptides prepared from tissue samples taken from fish fed on different diets were analyzed on a Q-Exactive HF-X mass spectrometer (Thermo Fisher Scientific) interfaced with an UltiMate 3000 UHPLC (Thermo Fisher Scientific) using data-independent acquisition (DIA) mode, employing the same column and gradient used for the library generation fractions. Twenty-one dynamic windows with precursor ions within isolation windows ranging from 22.0 to 589.0 *m*/*z* were subjected to fragmentation by HCD using a normalized collision energy of 27.5. The MS2 scan resolution was set at 30 k, and an AGC target value of 2 × 10^5^ was used.

### 2.9. Data Analysis

Raw files obtained from 17 fractions were analyzed using MSFragger version 21.1 to produce a spectral library using a barramundi FASTA file of 58,000 protein sequence entries downloaded in February 2024 from UniProt. The library generated by MSFragger from each tissue was used for DIA-NN (1.8.1) search for 16 samples of two different diets, with precursor and protein-level false discovery rates of no greater than 1% based on q-value calculations. The report.pg_matrix file generated by DIA-NN was used for statistical analysis by FragPipe Analyst (version 1.21) [17] using DIA type, Max-LFQ Intensity, variance stabilizing normalization, and maximum likelihood (MLE) imputation. Proteins detected in at least 3 out of 4 replicates per group with a *p*-value cutoff of *p* < 0.05 and a fold change of ≥1.3 and ≤0.76 were considered as differentially abundant proteins (DAPs). Since the sample group sizes were relatively small (less than 10), multiple comparison testing adjustment of *p*-values would likely result in increased Type-II errors, removing potentially important proteins from the results [18]. Benjamini–Hochberg adjusted *p*-values are provided in Supplementary Data S1.

Partial least squares discriminant analysis (PLS-DA) was performed using MetaboAnalyst 6.0 [19], and volcano plots of the three tissue samples were generated using Microsoft Excel. We employed the Limma software package (version 3.56.2) to perform statistical comparisons by fitting a linear model and applying empirical Bayes moderation to estimate variance. Protein abundances were log_2_-transformed, and group means were compared to calculated fold changes. This is equivalent to comparing geometric means on the raw scale but provides greater statistical robustness, particularly in the presence of missing values. Functional analysis of identified proteins, including gene ontology (GO) and KEGG pathways, was performed using the String database [20].

### 2.10. Parallel Reaction Monitoring Analysis

Parallel reaction monitoring (PRM) analysis was used for the validation of the label-free shotgun proteomics results to measure quantitative changes in specific proteins in the tissue of the barramundi fed on diet A and diet B. Skyline-daily was used to create an inclusion list of unique peptides, for each selected protein, with their mass-to-charge ratio [21]. The obtained PRM data were imported into Skyline and subject to quality control analysis. The sum of areas of all transition peaks for each peptide was collected and log2-transformed for four biological replicates of diet A and four biological replicates of diet B, and Student’s *t*-test analysis was used to compare protein abundance between the two diets.

### 2.11. Total Iron Analysis

Approximately 20 mg of liver and 50 mg of brain and intestine tissues from each biological replicate (*n* = 4 per tissue) were transferred to a microwave digestion tube (Multiwave 3000 Digestion Microwave, Anton Paar, North Ryde, Australia). A total of 5 mL of 69% nitric acid and 1 mL of 30% hydrogen peroxide was added to each. The microwave digestion settings were as follows: temperature, 180 °C; pressure, 500 psi; power, 1000–1800 W; ramp time, 20 min; and hold time, 20 min. After complete digestion and cooling for 10 min, the resultant solutions were transferred to 50 mL Falcon tubes, and the volume of the replicates was adjusted to 30 mL with Milli-Q water. Samples were diluted 9000-fold and analyzed by inductively coupled plasma-mass spectrometry (ICP−MS) with a limit of detection of parts per billion (ppb) [22].

## 3. Results and Discussion

### 3.1. Growth Performance

At the start of the trial, the barramundi averaged approximately 500 g in weight. At the end of the 12-week feeding trial, the average body weight reached 683 g for fish fed with Diet A and 684 g for those fed with Diet B, showing no significant difference between treatments. Full details of growth and feeding measurements are presented in Table 2.

Feed intake averaged 864 g for Diet A and was modestly lower at 828 g for Diet B; however, this variation was not statistically significant. In contrast, the FCR was significantly lower in fish fed Diet B (1.210) compared to those fed Diet A (1.265), indicating slightly better feed efficiency in the Diet B group.

### 3.2. Numerical Summary of Proteins Identified in Different Tissues

Data-independent analysis of quantitative proteomics was used to analyze four replicates of three tissue samples of the barramundi fed on two different commercial diets designated as diet A and B. The number of proteins identified and quantified in each replicate for diet A ranges from 2868 to 3342 for liver, 3270 to 3501 for brain, and 4295 to 4482 for intestine, and for diet B ranges from 2941 to 3356 for brain, 2051 to 3069 for liver, and 4471 to 4545 for intestine. Details of all these identified proteins are presented in Supplementary Data S1.

### 3.3. Estimating Effect Size of Within-Diet vs. Between-Diet Comparisons

In biological experiments, quantitative differences at the molecular level can stem from three main sources: biological variability, reflecting the natural diversity between organisms, subjects, or tissue samples; technical variability, which arises from the way we handle and measure those samples, including factors like instrumentation, assay methods, and preparation steps; and induced biological change, which is what we are trying to measure in order to indicate the effect of what external factors are imposed on the biological system [23].

To determine how much the observed changes in protein abundance were influenced by diet, we first performed within-diet comparisons as a control. This involved comparing results from four replicates of Diet A with results from a second set of four replicates of Diet A (A vs. A) and performing a similar analysis with results from two sets of four replicates of Diet B (B vs. B). These results were then compared to the variability observed between four replicates of Diet A and four replicates of Diet B (A vs. B). Expressing the number of differentially abundant proteins as a percentage of the total proteins identified in each comparison allows us to generate an approximate measure of effect size. If dietary composition had no significant effect, we would expect similar levels of effect size in both within- and between-diet comparisons. However, a noticeably higher percentage of differentially abundant proteins between diets would suggest that the dietary treatments had a greater effect size and thus a measurable impact on the proteomic profile.

Our analysis clearly showed that protein abundance differences were greater between the two diets than within the same diet, across all three tissues (see Table 3, Table 4 and Table 5). For example, in brain samples (Table 3), comparisons within the same diet showed only 6.01% (Diet A) and 2.41% (Diet B) of proteins were differentially abundant. But when comparing Diet A to Diet B, this jumped to 12.99%. A similar trend was seen in the liver in Table 4 (7.86% and 5.77% vs. 12.73%) and the intestine in Table 5 (2.20% and 5.21% vs. 16.59%).

The consistent differences across all tissues between the two diets suggest that what the fish were fed had a measurable effect on the proteomic profile in each organ.

The intra-group comparisons from all tissues yielded an average of 4.9% DAPs, which provides us with an estimate of the systemic noise since there should theoretically be very little difference between these samples. In contrast, the A vs. B comparisons yielded an average of 14.1% DAPs, which is nearly 3 times larger. Even though the diets were both commercially formulated and suitable for use in feeding trials, they still had a clear and consistent impact on the protein abundance profiles in the barramundi. By comparing replicates within the same diet, we were able to confirm that the differences seen between diets are not solely due to biological or random variation. This supports the idea that the proteins identified as differentially abundant between the two diets are altered in response to dietary changes and could serve as a useful index for evaluating the adaptive response of fish to the available nutrients.

### 3.4. Statistical Analysis of Protein Abundance in Brain

**Figure 1:**
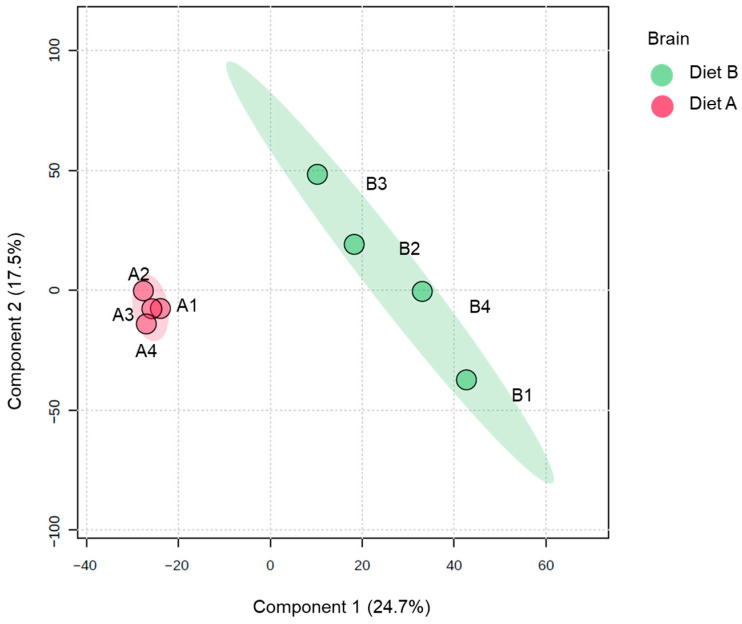
Partial least squares discriminant analysis (PLS-DA) of brain of the barramundi fed on diet A and diet B displayed at 95% confidence intervals highlights the clustering of the sample based on their response to different diets (*n* = 4).

Analysis of PLS-DA of the brain proteome data, which is used to provide a useful visual aid of the data distribution, revealed evidence of the clustering between protein abundance in the brain tissue samples (Figure 1). There is greater dispersion among diet B samples, suggesting higher inter-individual variability in response to diet B.

Full details of all these differentially abundant proteins are presented in Supplementary Data S2, along with a volcano plot that gives a comprehensive overview of protein abundance changes in the brain tissue. As shown in Table 3 a total of 302 proteins were decreased, and 205 proteins were increased in brain in fish fed on diet B.

#### 3.4.1. Differentially Abundant Proteins in Brain

Table 6 presents the top ten DEPs in the brain (presented in detail in Supplementary Data S2) when comparing the different diets, sorted by fold change. The brain regulates nearly all biological functions and behaviors, including eating and digestion. Its response to food and metabolic status, along with its ability to integrate information, influences an individual’s nutritional and emotional state. This interaction shapes the balance between hunger and satiety, the experience of pleasure, and the development of goal-directed behaviors [24].

##### Differential Abundance in Brain of Proteins Linked to Feeding Behavior

Peptidyl-prolyl cis-trans isomerase (PPIase) (EC 5.2.1.8) increased in the brain, which is involved in immune regulation [25]. This may suggest an adaptive response to different dietary compositions. Protein tyrosine phosphatase receptor type Nb (PTPRN) was increased in the brain. This family of proteins is highly abundant in brain regions like the hypothalamus and pituitary [26]. Notably, PTPRN is required for normal accumulation of dopamine and serotonin in the brain and is involved in insulin and peptide hormone secretion in endocrine cells [27].

Another protein increased in the brain is 3-Oxoacyl-(Acyl-Carrier-Protein) Synthase, which is an enzyme involved in the fatty acid biosynthesis pathway [28]. Even though the crude lipid ratio and fatty acids in both diets are similar, total diet composition might have impacted the brain by, for example, increasing the abundance of protein tyrosine phosphatase receptor type Nb to increase fatty acid synthesis (FAS) to compensate for the required fatty acids locally. Indeed, brain FAS is sensitive to diet and is regulated by nutrient availability that increases in states of excess energy or certain deficiencies [29]. FAS activity in the hypothalamus is known to influence feeding behavior by producing lipid signals that activate peroxisome proliferator-activated receptor alpha [29].

Several proteins involved in G-protein coupled receptor (GPCR) signaling were markedly lower in abundance in the brain of the fish fed on diet B. Notably, a guanine nucleotide-binding protein Gα subunit and another G-protein component were reduced, alongside a phosphatidylinositol 4,5-bisphosphate phosphodiesterase. Diet can strongly influence neuromodulatory systems; for example, diet imbalances or different sources might reduce the activity of neurohormonal pathways. GPCRs are central to regulating appetite, satiety, and metabolism [30]. These differences could have modulated neurohormonal pathways that regulate feeding behavior.

Cell adhesion molecule 1a (CAM1a) is another protein that was significantly reduced in the brain of fish fed diet B, implying reduced cell–cell adhesion in the brain. Cell adhesion molecules are crucial for synapse formation, maintenance, and neural plasticity [31]. A decrease in CAM could mean that fish fed on diet B had fewer or less stable synaptic contacts. One possible factor is the differences in the absorption of dietary fatty acids like omega-3 fatty acids, which are known to promote synaptic protein expression and plasticity [32].

#### 3.4.2. Functional Annotation of Enriched GO Terms in Brain

**Figure 2:**
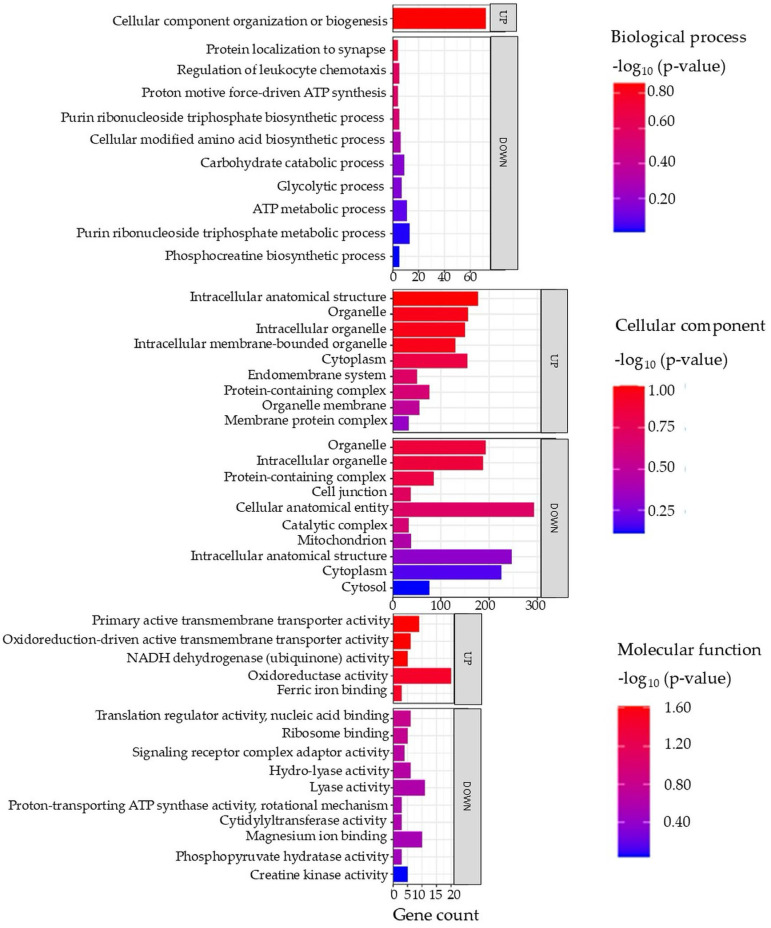
Top enriched GO terms in the DAPs of brain tissue from the barramundi. A higher −log_10_ (*p*-value) (red) indicates greater statistical significance, while lower −log_10_ (*p*-value) (blue) values indicate less significant enrichment. Up indicates increased abundance in the brain of fish fed on diet B, while Down indicates decreased abundance in the brain of fish fed on diet B. Gene count refers to the number of DAPs mapped to each GO term.

Gene ontology (GO) terms of the DAPs in the brain tissue are illustrated in Figure 2 (full details in Supplementary Data S3).

##### Enriched Biological Processes in Brain

Analysis of the enriched biological processes in brain tissue indicates that fish fed on diet B experience the downregulation of biological pathways such as the adenosine triphosphate (ATP) metabolic process and glycolytic and carbohydrate processes. The downregulation of these pathways points to reduced energy metabolism and cellular renewal. Given the comparable diets and absence of significant physiological differences, this effect may reflect subtle nutritional differences that are only detectable at the proteomic level [33].

The reduction in ATP-related processes often accompanies oxidative stress or inflammation, as organisms may divert energy toward repair and immune functions instead of growth [33]. Therefore, growth rate is suppressed, which is a well-known adaptation to poor nutrition. Inadequate nutrient intake in fish typically results in slower growth and smaller body size, as the fish conserves energy [34]. This is a common outcome of nutritional condition, reflected by poor weight gain and low feed efficiency in deficient diets [34]. Although no significant physiological differences were observed, the proteomic shifts in fish fed diet B may indicate subtle metabolic adaptations to the altered nutrient composition, representing an early molecular response to the available nutrients.

##### Enriched Cellular Components in Brain

There is an upregulation of cellular components, including the intracellular organelle membrane and cytoplasm, which might reflect cellular remodeling or immune activation. One plausible, albeit speculative, interpretation is the activation of autophagy. In fish, autophagy is induced during starvation as a survival mechanism, helping to recycle cellular constituents to provide nutrients to vital organs [35]. This process involves increased formation of double-membrane autophagosomes (derived from organelle membranes) that engulf cytoplasmic components for degradation, leading to the enrichment of membrane and cytoplasm-associated proteins. Such cellular remodeling can also tie into metabolic and adaptive responses in fish [36].

Meanwhile, downregulation of GO terms such as catalytic complex, mitochondrion, and cytosol might indicate a disruption in energy production, aligning with the depressed metabolic pathways noted above. This disruption is often linked to oxidative stress and iron deficiency. For instance, both oxidative stress and iron deficiency can impair mitochondrial function. Iron is a critical cofactor for many mitochondrial enzymes and the electron transport chain; iron deficiency is known to reduce oxidative phosphorylation capacity by limiting iron–sulfur cluster and heme synthesis in complexes I–III of the electron transport system [37]. In severe iron deficiency, mitochondria may even show structural abnormalities (e.g., loss of cristae) that compromise ATP production [37]. Proteomic evidence of mitochondrial protein downregulation in the brains of fish fed on diet B suggests a modulation of mitochondrial energy metabolism, potentially reflecting an adaptive response to subtle nutritional differences between the diets. Although physiological parameters did not differ significantly and the diets were compositionally comparable, these proteomic patterns indicate early molecular adjustments associated with variations in nutrient availability.

##### Enriched Molecular Functions in Brain

Upregulation was also observed of molecular functions including oxidative activity, nicotinamide adenine dinucleotide hydride (NADH) dehydrogenase, and active transmembrane transporter activity in the fish fed on diet B. These increases suggest that the cells were mounting a defensive response against oxidative responses. When oxidative response is elevated (for example, due to iron-mediated reactive oxygen species (ROS) or inflammation), organisms often induce antioxidant enzymes to counteract the damage. Indeed, studies on fish under fasting or stress show a significant upregulation of antioxidant defense proteins like superoxide dismutase (SOD), catalase (CAT), and glutathione peroxidase (GPX) [38]. In one study in Common Dentex (*Dentex dentex*) liver, starved fish increased their SOD, CAT, and GPX activities by 20–50% as a compensatory mechanism to neutralize the excess ROS, although oxidative damage still occurred [38]. The observed rise in oxidoreductase activity in fish fed on diet B aligns with this pattern.

#### 3.4.3. Enrichment of KEGG Pathways in Brain

**Figure 3:**
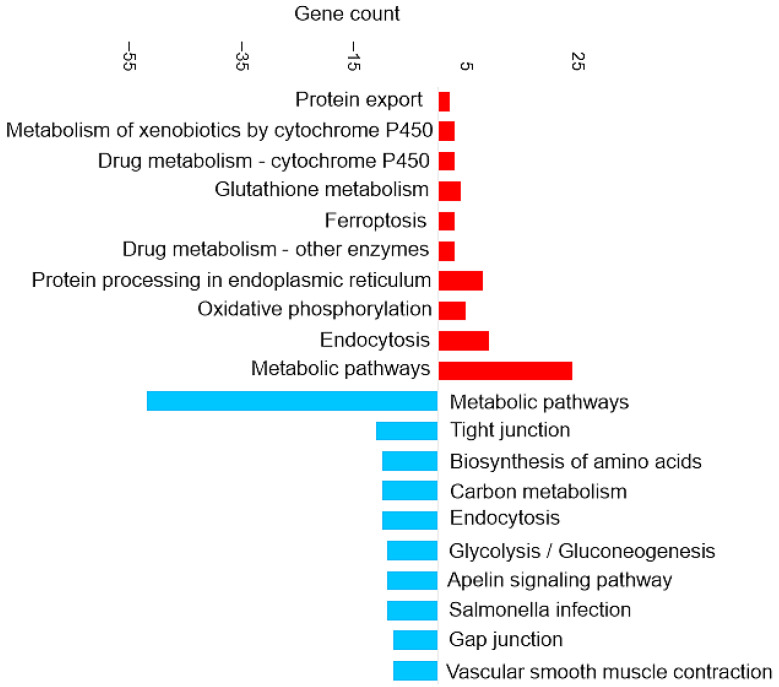
Pathway enrichment analysis based on the observed gene count of DAPs in the brain of the barramundi. Observed gene count refers to the number of DAPs mapped to each KEGG pathway. Red bars represent pathways enriched among proteins that increased in abundance in fish fed Diet B, whereas blue bars represent pathways enriched among proteins that decreased in abundance in Diet B.

Figure 3 shows the most enriched KEGG pathways of DAPs in brain tissue.

##### Enrichment in Brain of the KEGG Pathways for Oxidative Phosphorylation and Ferroptosis

Oxidative phosphorylation (shown in Figure 3) is a cellular process that utilizes the reduction of oxygen to produce high-energy phosphate bonds in the form of ATP. The electron transport chain uses NADH and flavin adenine dinucleotide (FADH2), which are generated from different catabolic processes within the cell [39]. However, electrons can leak from the cell and react with oxygen and produce superoxide anions. Excessive amounts of superoxide anions as a source of ROS can result in the oxidation of biological molecules such as lipids, proteins, and DNA [40].

Ferroptosis upregulation (shown in Figure 3) in the brain is related to the increase of ferritin. Ferroptosis was identified as an iron-dependent form of programmed cell death [41]. The unique process of ferroptosis includes the dysregulation of iron metabolism and the accumulation of ROS [42,43]. Some characteristics of ferroptosis are cytological changes, including decreased or vanished mitochondria cristae, a ruptured outer mitochondrial membrane, and a condensed mitochondrial membrane [44].

In the present study, although ferroptosis-related proteins were upregulated in fish fed on Diet B, there were no significant differences in physiological parameters or feed intake between the two dietary groups. This suggests that the observed ferroptosis activation may represent an adaptive regulatory response to subtle nutritional differences, rather than a pathological outcome. Such molecular adjustments likely reflect normal cellular mechanisms maintaining iron and redox homeostasis under slightly varying dietary conditions.

##### Enrichment in Brain of the KEGG Pathways for Glutathione Metabolism and Antioxidant Response

The glutathione metabolism KEGG pathway was also upregulated (shown in Figure 3), which includes the increase of glutathione transferase (EC 2.5.1.18). The upregulation of glutathione metabolism may represent a specific compensatory mechanism to counteract and balance ferroptosis signaling. Glutathione (GSH) is a critical endogenous antioxidant found in all eukaryotic cells [45]. Increasing the supply of cysteine or its precursors via oral or intravenous administration enhances GSH synthesis and prevents GSH deficiency in humans and animals under various nutritional and pathological conditions [46]. Cysteine depletion can trigger iron-dependent nonapoptotic cell death—ferroptosis [47]. Even though cysteine itself is not an essential amino acid, methionine is the metabolic precursor for cysteine [48]. The higher essential amino acid content of methionine in diet B may also have induced compensatory responses in the brain, as reflected by the upregulation of glutathione metabolism. This shift likely represents an antioxidant strategy to counter ROS accumulation.

##### Enrichment in Brain of the KEGG Pathways for Appetite Regulation and Digestive Response

The downregulation of certain KEGG pathways in the brain, such as apelin signaling (shown in Figure 3), may represent compensatory mechanisms attempting to mitigate the effects of dietary composition and slightly lower food intake. Apelin is a food intake-regulating peptide [49]. Guanine nucleotide-binding protein subunit alpha is one of the proteins involved in the apelin signaling pathway that decreased. Research on common crab has shown that Pyr-apelin-13 supplementation can promote food intake and growth by regulating the mRNA expression levels of key genes [50]. Apelin has been shown to influence appetite by interacting with regulatory signals such as leptin and ghrelin. These observations suggest that apelin might play a role in regulating feeding behavior and maintaining energy homeostasis [49].

It has been shown previously that infusion of apelin reduced the juice volume, protein, and trypsin outputs in a dose-dependent manner [49]. Trypsin is one of the serine proteinases in fish viscera that breaks down the protein [51]. For example, rats fed raw soybeans showed decreased body weight and increased pancreas weight due to the presence of trypsin inhibitors in the soybean since soybean can increase the release of cholecystokinin (CCK) [52]. Trypsin as a digestive proteinase has been shown to reduce the release of CCK [52], which is a peptide hormone and a neurotransmitter expressed mainly in a subpopulation of small intestinal endocrine cells (I-cells) and in cerebral neurons [53]. CCK is believed to make the rat feel full, and higher levels of CCK might lead to eating less [52]. In this study, the nutritional profile for both diets contains similar levels of faba bean, which has trypsin inhibitor activity [54]. The KEGG pathway analysis suggests that the dietary composition may include compounds that impact the digestibility or bioavailability of the nutrients by reducing the trypsin activation.

### 3.5. Statistical Analysis of Protein Abundance in Liver

**Figure 4:**
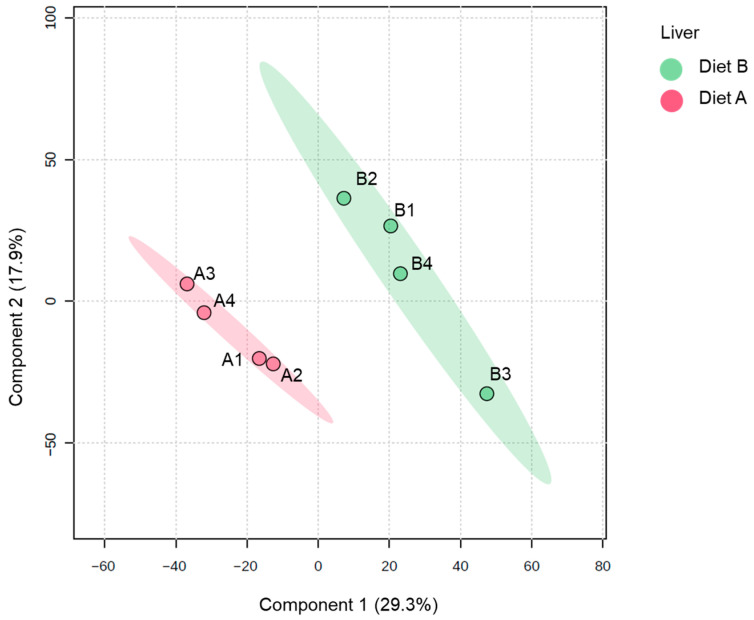
Partial least squares discriminant analysis (PLS-DA) of the liver of the barramundi fed on diet A and diet B displayed at 95% confidence intervals highlights the clustering of the sample based on their response to different diets (*n* = 4).

The PLS-DA plot of proteins identified in the liver samples (Figure 4) shows a distinct separation between the two dietary groups along the first two principal components. The ellipses representing the 95% confidence intervals are well separated, indicating a high degree of discriminative power between diet A and diet B at the proteomic level in the liver. Samples from diet A are tightly clustered together and distinctly separated from the diet B samples, which also form a cohesive cluster. The results underscore that the fish liver proteome is highly responsive to dietary composition, which induces marked shifts in hepatic protein abundance profiles [55,56,57].

For liver tissue, 231 proteins were decreased, and 235 proteins were increased in the liver of fish fed on diet B, as shown in Table 4. Full details of all DAPs in the liver are presented in Supplementary Data S4, along with a volcano plot visualizing changes in protein abundance. Notably, these diet-induced proteomic changes are similar to a previous study on spotted scat (*Scatophagus argus*), where dietary supplementation of oil applied showed a significant differential protein abundance in the liver between groups [58].

#### 3.5.1. Differentially Abundant Proteins in Liver

Table 7 presents the top 10 DAPs, sorted by fold change, in the liver of fish when comparing the different dietary formulations. The liver is the primary site where absorbed nutrients are processed after leaving the gut. It plays a central role in metabolizing carbohydrates, fats, and proteins; activating and storing vitamins; and detoxifying and eliminating both internal and external compounds [59].

##### Differential Abundance in Liver of Proteolytic Enzymes

The synthesis of pancreatic enzymes and their secretion into the duodenum are essential for digestion of dietary macromolecules and absorption of nutrients [60]. In particular, the activities of proteolytic enzymes are modified either by the protein level and the source of protein in the diet or by food intake [61,62]. Our results are similar to these findings, as Table 7 shows an increase in the amount of pancreatic elastase II and chymotrypsin. Elastase II, which is closely related to the chymotrypsin family [63], exhibits a broad specificity for substrates containing medium-to-large hydrophobic amino acids in the P1 position [60]. Diet B includes a higher proportion of hydrolyzed feather meal. Feather meal includes a high proportion of keratin, which has a high concentration of hydrophobic amino acids [64]. Keratin is resistant to digestion by the proteases pepsin or trypsin [65]. The increase in abundance of proteases expressed in the liver of fish fed on diet B could potentially be an adaptive response to these compositional differences to regulate the digestion of the fish.

##### Differential Abundance in Liver of Proteins Linked to RNA Processing and Cellular Response

RNA helicase (DDX5) is another protein that was increased in the liver with a fold change of 4.86 (Table 7). DDX5 (also known as p68) is one of the prototypic members of the DEAD box family of RNA helicases. DDX5 and related DDX17 (p72) are involved in a variety of cellular processes, including transcription, pre-mRNA and rRNA processing, alternative splicing, and miRNA processing, and they are also dysregulated in a range of cancers [66,67,68,69]. The RNA helicase DDX5 functions as a coactivator of the tumor suppressor protein p53, playing a crucial role in the DNA damage response by facilitating the transcription of genes like CDKN1A (p21), which are essential for cell cycle arrest [70,71]. However, studies have shown that while DDX5 is necessary for p53-dependent induction of p21 and subsequent cell cycle arrest, it does not participate in the activation of pro-apoptotic genes [71]. We speculate that DDX5 may influence the cellular decision to undergo cell cycle arrest rather than apoptosis in response to DNA damage as a result of conditions triggered by ferroptosis, thereby contributing to cell survival under certain conditions. Lysozyme g was also decreased in the liver of fish fed on diet B that contains more methionine, which is an important secretory innate immune system component [72]. Lysozyme activities were influenced when *Scophthalmus maximus* were stressed [73]. In European seabass, fish fed with methionine supplementation showed an increase in the level of lysozyme, and a higher survival was observed in fish fed with supplemented diets [74].

#### 3.5.2. Functional Annotation of Enriched GO Terms in Liver

**Figure 5:**
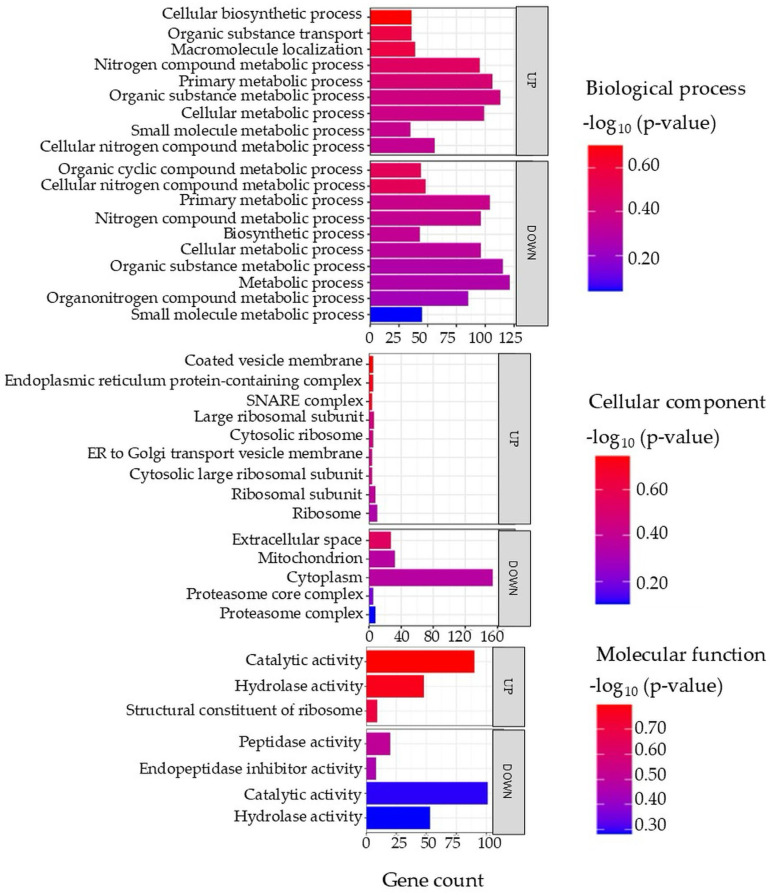
Top enriched GO terms in the DAPs of the liver of the barramundi. A higher −log_10_ (*p*-value) (red) indicates greater statistical significance, while lower −log_10_ (*p*-value) (blue) values indicate less significant enrichment. Up indicates increased abundance in the liver of fish fed on diet B, while Down indicates decreased abundance in the liver of fish fed on diet B. Gene count refers to the number of DAPs mapped to each GO term.

Gene ontology (GO) terms of the DAPs in the liver tissue are illustrated in Figure 5, with full details provided in Supplementary Data S5. In the biological process category, both increased and decreased proteins were predominantly associated with metabolic processes, including cellular metabolic processes, small molecule metabolic processes, nitrogen compound metabolism, and biosynthetic pathways. The cellular component analysis showed that increased proteins were enriched in ribosomal subunits, endoplasmic reticulum protein-containing complexes, and vesicle transport components. In contrast, decreased proteins were linked to the cytoplasm, proteasome complex, mitochondria, and extracellular space. In the molecular function category, increased proteins were associated with catalytic and hydrolase activity as well as ribosome structural components, while decreased proteins were related to peptidase and endopeptidase inhibitor functions.

#### 3.5.3. Enrichment in Liver of the KEGG Pathway for Ferroptosis

**Figure 6:**
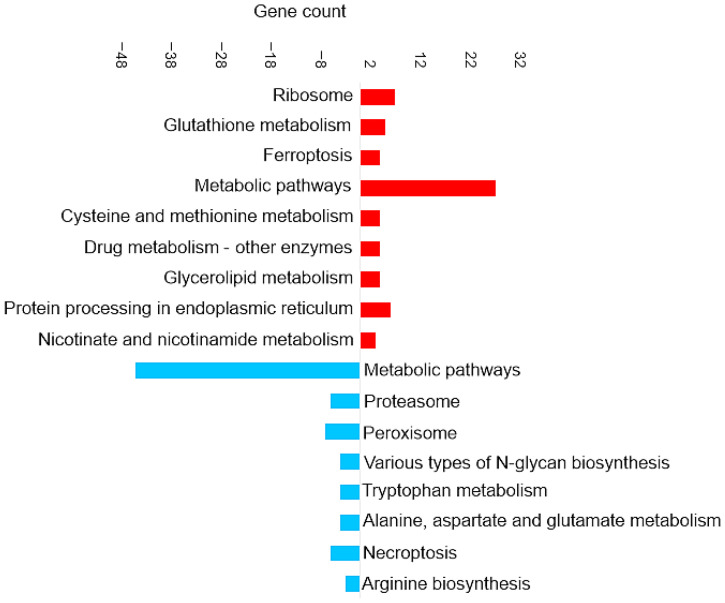
KEGG pathway enrichment analysis based on the observed gene count of DAPs in the liver of the barramundi. Observed gene count refers to the number of DAPs mapped to each KEGG pathway. Red bars represent pathways enriched among proteins that increased in abundance in fish fed Diet B, whereas blue bars represent pathways enriched among proteins that decreased in abundance in Diet B.

Figure 6 shows the most enriched KEGG pathways of DAPs in the liver. Ferroptosis and glutathione metabolism are two of the upregulated pathways in liver tissue, as shown in Figure 6. This is similar to the results discussed in the brain tissue in Section 3.4.3. Proteins involved in the ferroptosis pathway include glutathione peroxidase, glutathione synthetase, Lys phosphatidylcholine acyltransferase 3, and one uncharacterized protein (for details, see Supplementary Data S5).

### 3.6. Statistical Analysis of Protein Abundance in Intestine

**Figure 7:**
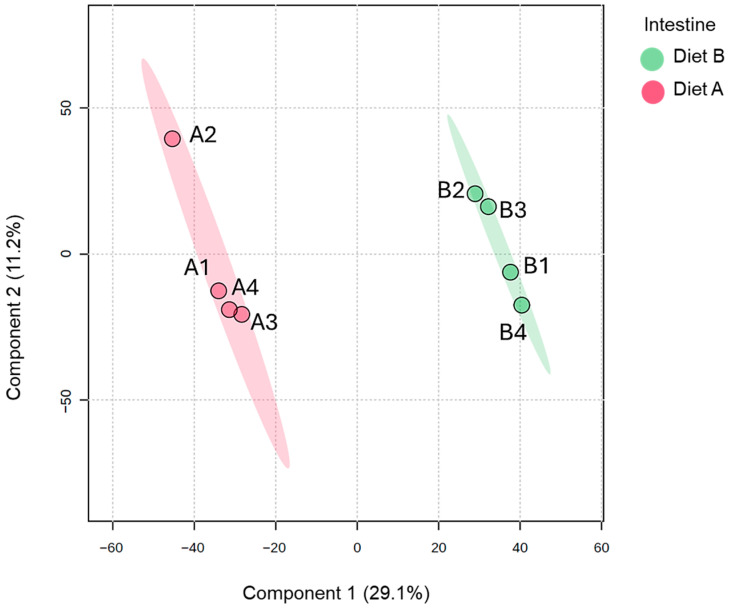
Partial least squares discriminant analysis (PLS-DA) of the intestine of the barramundi fed on diet A and diet B displayed at 95% confidence intervals highlights the clustering of the sample based on their response to different diets (*n* = 4).

The PLS-DA analysis of the intestine samples (presented in Figure 7) shows a similar separation between the dietary groups as was observed for brain and liver tissues, although the replicates are not as tightly clustered together.

As shown in Table 5, 349 proteins were increased, and 485 proteins were decreased in the intestine of fish fed on diet B when compared with diet A. Full details are presented in Supplementary Data S6, along with a volcano plot visualizing the protein abundance distribution. The observed fold change values in the intestine are higher than for the other tissues, which may reflect the role of the intestine in nutrient absorption and barrier functions, with involvement in metabolic regulation, indicative of tissue-specific sensitivity to dietary interventions.

#### 3.6.1. Differentially Abundant Proteins in Intestine

The intestine is responsible for the enzymatic breakdown and uptake of macronutrients such as proteins, lipids, and carbohydrates, but also for the absorption of micronutrients, including vitamins and minerals [75]. The intestinal epithelium is highly specialized, containing a variety of transporters, enzymes, and structural proteins that regulate barrier integrity and nutrient passage [76]. Fish intestines are vital for immunological response, nutrition absorption, and environmental interactions [77]. Proteomic profiling of intestinal tissues is therefore a powerful approach to assessing how dietary interventions as an environmental factor affect gut function and nutrient utilization. Table 8 presents the top ten proteins as sorted by fold change, which were increased and decreased in the intestine tissue of fish fed on diet B compared with fish fed on diet A.

##### Differential Abundance in Intestine of Proteins Linked to Immune Signaling and Lipid Metabolism

NACHT domain-containing protein increased with a fold change of 5.29 (Table 8). This protein is found in eukaryotic pattern recognition receptors involved in promoting anti-microbial defense, mediating their oligomerization into immune signaling complexes. The NACHT module is an oligomerization domain that facilitates assembly of multiprotein, immune-signaling complexes in response to microbial ligands or disrupted physiological processes [78]. Another protein increased is apolipoprotein Ea (ApoE) with a 5.16-fold change. ApoE is also implicated in infections with herpes simplex type-1, hepatitis C, and human immunodeficiency viruses [79]. Oxidative responses have been shown to augment ApoE secretion from adipocytes, and ApoE overexpression protected cells from hydrogen peroxide-induced damage [80].

Fatty acid-binding protein decreased with a fold change of 0.31 (Table 8). Fatty acid-binding protein is essential for digesting and absorbing dietary fats by transporting bile acids in intestinal cells. It helps break down cholesterol and may be linked to bile acid transport in the intestine [81]. High-fat feeding of intestinal fatty acid-binding protein in mice resulted in reduced weight gain and fat mass relative to wild-type mice [82]. The mRNA levels of fatty acid synthase and sterol regulatory element-binding protein-1 (SREBP1) genes increased with increasing dietary methionine in juvenile cobia [83].

#### 3.6.2. Functional Annotation of Enriched GO Terms in Intestine

**Figure 8:**
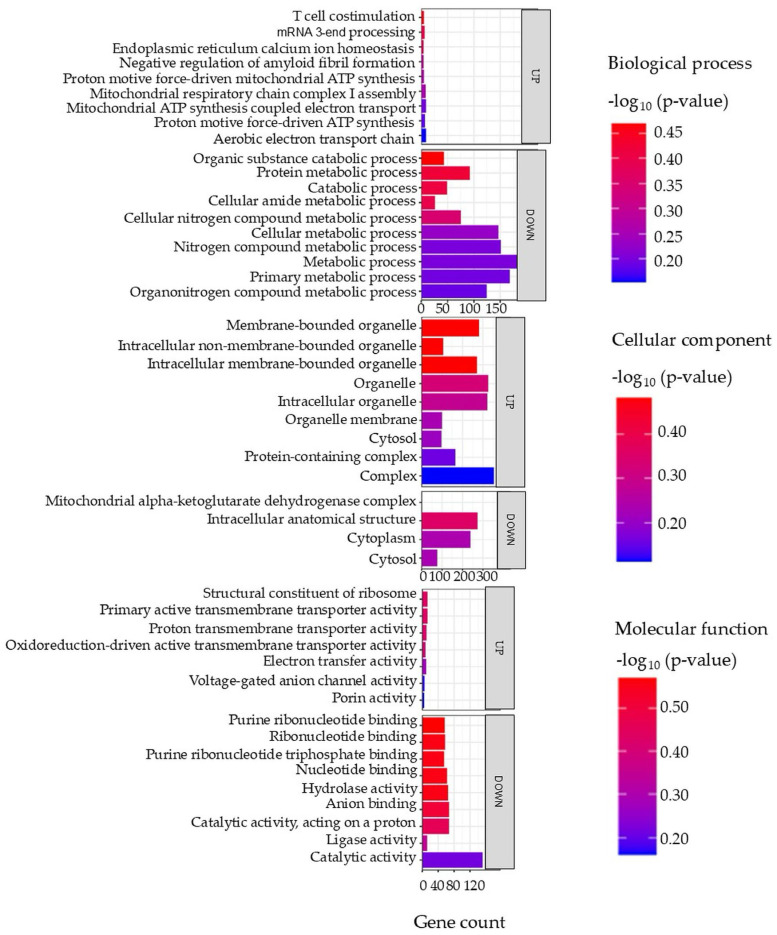
Top enriched GO terms in the DAPS of the intestine of the barramundi. A higher −log_10_ (*p*-value) (red) indicates greater statistical significance, while lower −log_10_ (*p*-value) (blue) values indicate less significant enrichment. Up indicates increased abundance in the intestine of fish fed on diet B, while Down indicates decreased abundance in the intestine of fish fed on diet B. Gene count refers to the number of DAPs mapped to each GO term.

Figure 8 presents the most enriched GO terms represented within the set of DAPs from intestinal tissue.

Among the upregulated proteins, pathways associated with mitochondrial energy production were enriched, including the aerobic electron transport chain and proton motive force-driven ATP synthesis, suggesting an increased mitochondrial activity and energy demand. Correspondingly, upregulated cellular components were primarily linked to the mitochondrial membrane, protein-containing complexes, and membrane-bounded organelles, reinforcing the role of mitochondria in the observed metabolic response.

In contrast, proteins that decreased in abundance were associated with metabolic and catabolic processes, particularly those involving nitrogen-containing compounds, protein metabolism, and primary cellular metabolism. These trends were further supported by the enrichment of cytoplasmic and intracellular organelle components, suggesting a suppression of general metabolic pathways.

At the molecular function level, increased proteins were associated with transmembrane transporter activities, including those involved in electron and proton transport, aligning with enhanced mitochondrial and membrane transport function. Conversely, downregulated functions included catalytic activity, nucleotide binding, and hydrolase activity, indicating a general downregulation of biosynthetic and enzymatic processes.

Because ferroptosis recurs in all tissues and promotes ROS generation, it is likely tied to the shifts observed in metabolic proteins. Cells produce ROS in multiple locations, including cytoplasm, membranes, ER, mitochondria, and peroxisomes [84], but mitochondria are the biggest source, creating about 90% of the total ROS [85].

#### 3.6.3. Enrichment of KEGG Pathways in Intestine

**Figure 9:**
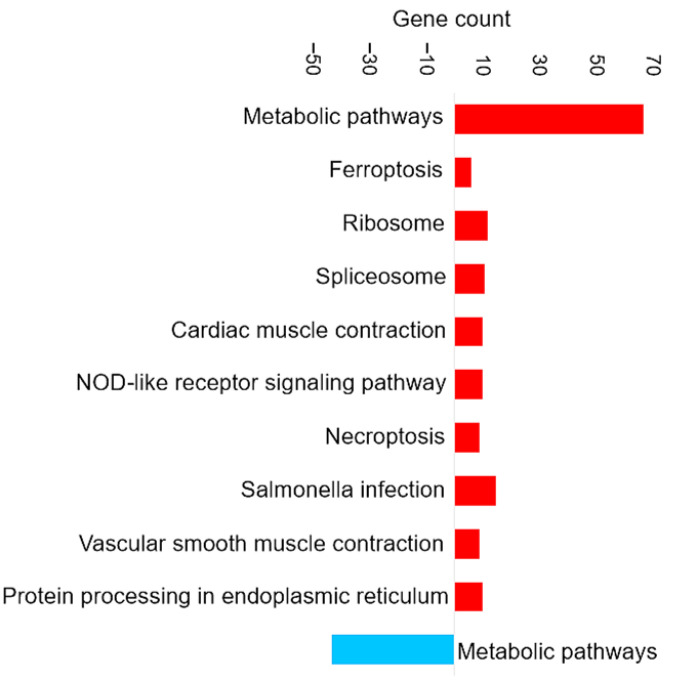
KEGG pathway enrichment analysis based on the observed gene count of DAPs in the intestine of the barramundi. Observed gene count refers to the number of DAPs mapped to each KEGG pathway. Red bars represent pathways enriched among proteins that increased in abundance in fish fed Diet B, whereas blue bars represent pathways enriched among proteins that decreased in abundance in Diet B.

Figure 9 shows the most enriched KEGG pathways in the set of DAPs in the intestine (full details in Supplementary Data S7). Upregulated pathways included ferroptosis, as was also observed in brain and liver tissue. The most prominent enrichment was of metabolic pathways, which was primarily driven by proteins involved in amino acid biosynthesis and glutathione metabolism. Proteins associated with amino acid biosynthesis that were increased in abundance in the intestine included phosphoserine aminotransferase, ribulose-phosphate 3-epimerase, phosphoglycerate mutase, and argininosuccinate lyase.

##### Enrichment in Intestine of KEGG Pathways for Amino Acid Biosynthesis

Phosphoserine aminotransferase is part of the phosphorylated pathway of serine biosynthesis [86]; serine is a non-essential proteinogenic amino acid that serves as a precursor for glycine, tryptophan, and cysteine [87]. In addition to its role in protein synthesis, serine is a key intermediate in the production of phospholipids and nucleobases [88]. Under environmental conditions in plants, the activity of this phosphorylated pathway is alleviated, which suggests that supplying serine is important for non-photosynthetic cells under harsh conditions [89].

Argininosuccinate lyase is involved in the urea cycle, resulting in the production of arginine and fumarate [90]. Fish have particularly high requirements for dietary arginine because it is abundant in proteins as a peptide-bound amino acid and in tissue fluid as phosphoarginine, a major reservoir of ATP, while its de novo synthesis is limited or even completely absent [91]. Arginine also plays a crucial role in regulating the endocrine system, which releases hormones directly into the bloodstream [91,92]. Although feed intake was slightly lower in diet B, this did not appear to limit amino acid availability. Arginine may regulate endocrine pathways and could have contributed to the improved feed conversion ratio observed. In addition, amino acid metabolism is central to iron and lipid homeostasis and redox balance and thus may play a role in modulating ferroptosis [93].

### 3.7. PRM Validation

**Figure 10:**
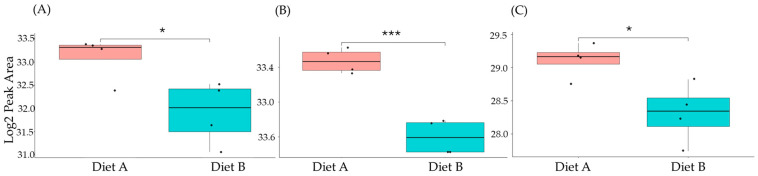
PRM validation of three proteins changed in abundance in response to dietary composition in brain tissue in the barramundi. (**A**) = guanine nucleotide binding protein (G protein), (**B**) = ATP synthase subunit beta (EC 7.1.2.2), (**C**) = ADP ribosylation factor like GTPase 3b. An asterisk (*, **, ***) indicates a statistically significant difference between diet A and diet B, according to a Student’s *t*-test (*p*-value ≤ 0.05, ≤ 0.01, ≤ 0.001).

**Figure 11:**
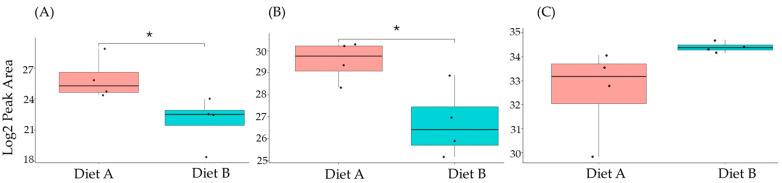
PRM validation of two proteins changed in abundance in response to dietary composition in liver tissue in the barramundi. (**A**) = lysozyme g, (**B**) = C1q domain-containing protein, (**C**) = pancreatic elastase. An asterisk (*, **, ***) indicates a statistically significant difference between diet A and diet B, according to a Student’s *t*-test (*p*-value ≤ 0.05, ≤ 0.01, ≤ 0.001).

**Figure 12:**
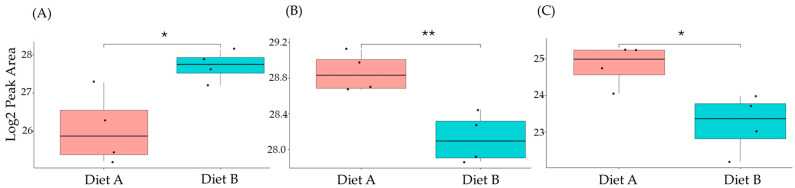
PRM validation of three proteins changed in abundance in response to dietary composition in intestine tissue in the barramundi. (**A**) = apolipoprotein Ea, (**B**) = non-specific serine/threonine protein kinase, (**C**) = phosphatidylinositol-4,5-bisphosphate 3-kinase. An asterisk (*, **, ***) indicates a statistically significant difference between diet A and diet B, according to a Student’s *t*-test (*p*-value ≤ 0.05, ≤ 0.01, ≤ 0.001).

A series of PRM experiments were performed to validate the results from label-free shotgun proteomics analysis of tissue from the barramundi. The PRM results indicated that the differential changes in abundance of nine selected proteins measured by PRM, as shown in Figure 10, Figure 11 and Figure 12, agreed with the label-free shotgun proteomics results. Quantification was based on the integrated peak areas of the most intense y- and b-type fragment ions selected by Skyline from the spectral library for each peptide. The list of monitored precursor ions, fragment ions (transitions), and their corresponding *m*/*z* values is provided in Supplementary Data S8.

This included guanine nucleotide binding protein (G protein), ATP synthase subunit beta (EC 7.1.2.2), and ADP ribosylation factor-like GTPase 3b that were decreased significantly in the brain of fish fed on diet B in abundance (Figure 10). In the liver, Lysozyme g and C1q domain-containing protein were decreased significantly in fish fed on diet B. Pancreatic elastase protein increased in the liver, but it was not significant (Figure 11). In the intestine, Apolipoprotein E increased significantly in the fish fed on diet B, and non-specific serine/threonine protein kinase and phosphatidylinositol-4,5-bisphosphate 3-kinase were decreased significantly in abundance (Figure 12).

### 3.8. Iron Analysis

Since the proteomic results in all three tissues demonstrated the upregulation of the ferroptosis signaling pathway, total iron analysis was performed to validate the quantitative proteomic results by confirming the accumulation of iron in the selected tissues, including brain, liver, and intestine. It has been shown that iron overload disrupts iron homeostasis, induces oxidative responses, promotes ferroptosis, and compromises the functional competence of chondrocytes [94].

#### 3.8.1. Total Iron Analysis in Brain Tissue

**Figure 13:**
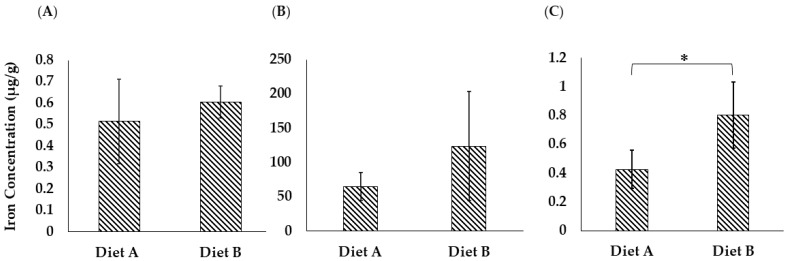
Total iron concentration (µg/g) in (**A**) brain tissue, (**B**) liver tissue, and (**C**) intestine tissue of the barramundi fed on diets A and B. Bars represent the standard deviation (*n* = 4). An asterisk (*) indicates a statistically significant difference between groups as determined by a Student’s *t*-test (*p* ≤ 0.05).

Iron is a crucial micronutrient in fish physiology, especially for brain function [95]. Iron is a vital functional component of many proteins and enzymes in organisms. It is actively involved in a series of physiological processes, including erythropoiesis, oxygen delivery, energy metabolism, and immune response [96]. The ferroptosis pathway was upregulated in the brain of the fish fed on diet B, as discussed in Section 3.4.3. As shown in Figure 13A, the concentration of iron measured in the brain of the fish fed on diet B was higher on average (B = 0.605 ± 0.073, A = 0.514 ± 0.196 µg/g), although the difference was not statistically significant due to the variability between biological replicates (Supplementary Data S9).

However, elevated brain iron levels must be interpreted with caution, as even small changes can be important. While sufficient iron is necessary for optimal brain development and performance, excessive iron accumulation can lead to oxidative responses due to the Fenton reaction, producing free radicals that damage neural tissue [97]. In this study, it is important to note that the growth parameter differences between two diets were not significant, and the FCR was significantly lower for diet B. This suggests that changes in the abundance of the proteins in the fish tissues are a physiological response to different nutrient availability, not necessarily a stress response.

#### 3.8.2. Total Iron Analysis in Liver Tissue

As shown in Figure 13B, the concentration of iron in the liver of the barramundi fed on diet B is 123.86 ± 79.14 µg/g, while for diet A it is lower at 64.96 ± 20.10 µg/g. However, even though the average values are clearly different, the result is not statistically significant due to the variability between biological replicate analyses. The proteomic data support the observed difference in iron levels, as it shows clear changes in the ferroptosis pathway (Figure 6).

#### 3.8.3. Total Iron Analysis in Intestine Tissue

As shown in Figure 13C, the iron concentration in the intestinal tissue of the barramundi was noticeably higher in fish fed on diet B (0.803 ± 0.227 µg/g) compared to those fed on diet A (0.425 ± 0.132 µg/g), and this difference was statistically significant. This finding suggests that while iron may be present in the intestinal lumen of fish fed on diet B, its absorption or subsequent transport into circulation might be less efficient than in fish fed on diet A. In contrast, the lower intestinal iron levels in fish fed on diet A may reflect more efficient uptake and systemic transport, potentially supported by components in the diet containing more land animal proteins.

Several factors are known to affect Fe absorption, including the amount and chemical form of Fe, age and Fe status of the animal, physiological conditions of the gastrointestinal tract (e.g., pH), and other dietary components (e.g., phytic acid, ascorbic acid, citrate) [98]. In fish, ferric iron (Fe^3+^) is released from food in the acidic environment of the stomach and binds to mucin, which may help keep it soluble in the small intestine. Although the exact mechanism of iron absorption in marine fish remains unclear, it is believed that mucus secretion plays a key role in maintaining iron solubility. However, metal–mucus complexes, dietary reducing agents like ascorbic acid, and other gut conditions may help enhance iron solubility and absorption [95].

#### 3.8.4. Summary of Results and Future Directions

Aquaculture faces ongoing pressure to identify alternative ingredients that support more sustainable feed development. As the industry shifts toward using alternative protein sources to reduce reliance on fishmeal, it becomes increasingly important to understand how these changes may affect fish health and overall well-being [99].

By examining the brain, liver, and intestine, we found that even small differences in dietary protein sources can lead to noticeable changes in proteins related to metabolism, nutrient processing, and immune function. These results are aligned with a previous study performed on the rainbow trout on the transcriptomic level, in which GO and pathway analysis of the corresponding genes revealed that nutrition affects pathways of neuroendocrine peptides in the brain, and in the liver, pathways mediating intermediary metabolism, xenobiotic metabolism, proteolysis, and cytoskeletal regulation of cell cycle [100].

Key appetite-regulating signals were altered in the brain. For example, components of the apelin signaling pathway were downregulated in fish fed on diet B. Apelin peptides are known to act as appetite stimulants in vertebrates; in mammals, apelin increases food intake and improves energy balance [101], and recent experiments suggest a similar appetite-promoting role in fish [101].

In the liver, changes in the diet influenced digestive enzymes and RNA-processing proteins, such as elastase II, chymotrypsin, and DDX5. It is well known that diet quality and ingredient type can influence hepatic metabolism in fish. For example, completely replacing fishmeal with plant proteins has been shown to alter hepatic proteomic profiles and induce metabolic pathways in other species [100]. One interesting observation was the activation of proteins linked to ferroptosis, which is a type of cell death triggered by too much iron and oxidative response [102].

In the intestine, proteins involved in fat transport and general metabolism were reduced, suggesting a shift in how nutrients were being absorbed and processed. Some stress-related proteins, like NACHT domain-containing proteins and ApoE, were also elevated, reflecting normal physiological adjustments to varying nutrient availability rather than a sign of immune activation. Although fish fed on diet B showed upregulation of ferroptosis and higher iron content in the intestine, it seems upregulation of glutathione metabolism as an antioxidant mediated the ferroptosis [103]. The observed changes at the protein level may also reflect regulation at the proteoform level, as proteins can exist in multiple modified or processed forms that influence their biological function [104].

Altogether, this research highlights how what fish eat, despite having comparable feed intake, influences their biology at the protein level. When the immune system is activated, it demands specific nutrients, which can lead to competition between what is needed for basic maintenance, immune function, and growth-related protein deposition. This is especially important in aquaculture, where nutrition plays a key role in fish health. As feed is the largest cost in aquaculture production, optimizing diet formulation is essential for both animal welfare and economic sustainability [99].

## 4. Conclusions

This study presents the first detailed quantitative proteomic analysis of the barramundi (*Lates calcarifer*) using a DIA-MS-based approach. It aimed to understand how two commercial diets with the same crude protein and fat content but different ingredient compositions affect different tissues of the fish at a molecular level. In this study, the growth performance between fish fed on two diets was not significant, and the FCR was significantly lower for fish fed on diet B. In the brain, we observed changes in energy production and oxidative response pathways, glutathione metabolism, and ferroptosis. In the liver we observed activation of antioxidant and ferroptosis-related pathways, which is consistent with a potential adaptive response. In the intestine, there was increased activity in amino acid biosynthesis, glutathione metabolism, and mitochondrial energy production. Proteins linked to serine and arginine production were more abundant, possibly helping the fish to adapt to different nutrient compositions.

The results of quantitative proteomic analysis were confirmed using PRM analysis of a selected subset of proteins. The proteomic results showed ferroptosis was significantly altered in all three tissues, which led us to perform direct analysis of iron levels. The iron analysis was only statistically significant in the intestine but demonstrated a higher level of iron in all three tissues for the fish fed on diet B.

## 5. Limitations

Although this study provides valuable insight into the tissue-specific proteomic responses of *Lates calcarifer* to two commercial diets, several limitations should be acknowledged. The proteomic analysis focused on total protein abundance, and post-translational modifications or specific proteoforms were not directly assessed. These modifications may play critical roles in metabolic regulation and warrant further investigation using top-down or targeted proteomic approaches. While this study identified molecular signatures associated with dietary composition, complementary measurements such as metabolomics or transcriptomics could provide additional layers of evidence linking molecular changes to physiological outcomes. Finally, the feeding trial was conducted under controlled laboratory conditions, which may not fully represent environmental variations in commercial aquaculture systems. Future work should evaluate how these molecular responses translate under production-scale settings.

## Figures and Tables

**Table 1:** Relative amounts of different components of the two diets.

Component	Relative Difference (A vs. B)	Interpretation
Wild catch sardine fish meal	0.4×	Diet A contains 60% less fishmeal (0.4× of Diet B)
Rendered poultry meal	2.1×	Diet A contains slightly more than twice as much poultry meal
Hydrolyzed feather meal	0.67×	Diet A contains 33% less feather meal
Blood meal	1.67×	Diet A contains 67% more blood meal
Phospholipids	0.61×	Diet A contains 39% less phospholipids
Methionine	0.78×	Diet A contains 22% less methionine
Iron	1.34×	Diet A contains 34% more iron

**Table 2:** Fish growth and performance measurements.

	Diet A	Diet B
Initial body weight (g/fish)	513.92 ± 0.472 *	513.49 ± 0.314
Final body weight (g/fish)	1197.17 ± 5.716	1197.21 ± 11.35
Specific growth rate (%/fish/day)	1.007 ± 0.00005	1.008 ± 0.0020
Gain (g/fish)	683.25 ± 5.26	683.72 ± 11.66
Feed intake (dry matter g/fish)	864.35 ± 8.87	827.85 ± 10.00
Food conversion ratio	1.265 ± 0.005	1.211 ± 0.012

* All values presented as mean ± standard error.

**Table 3:** Comparison of proteins from brain samples within and between diets.

Diet	Total	Increased	Decreased	Total Changed	Percentage
A vs. A	3889	101	133	234	6.01%
B vs. B	3817	31	61	92	2.41%
A vs. B	3901	205	302	507	12.99%

**Table 4:** Comparison of proteins from liver samples within and between diets.

Diet	Total	Increased	Decreased	Total Changed	Percentage
A vs. A	3649	144	143	287	7.86%
B vs. B	3530	94	110	204	5.77%
A vs. B	3660	235	231	466	12.73%

**Table 5:** Comparison of proteins from intestine samples within and between diets.

Diet	Total	Increased	Decreased	Total Changed	Percentage
A vs. A	5030	48	63	111	2.20%
B vs. B	5003	135	126	261	5.21%
A vs. B	5025	485	349	834	16.59%

**Table 6:** Top ten DAPs in brain tissue.

	Protein ID	Protein Name	Fold Change *
**Increased abundance in diet B**			
1	A0A4W6EX76	Transporter	7.49
2	A0A4W6ER29	Ferritin	4.61
3	A0A4W6D6I8	Peptidyl-prolyl cis-trans isomerase (PPIase)	3.64
4	A0A4W6D1Z6	Adhesion G protein-coupled receptor L2b, tandem duplicate 1	3.54
5	A0A4W6EE98	Myosin X, like 1	3.42
6	A0A4W6ETZ5	Protein tyrosine phosphatase receptor type Nb	3.42
7	A0A4W6FSI9	LIM zinc-binding domain-containing protein	3.39
8	A0A4W6EUJ3	3-oxoacyl-(acyl-carrier-protein) synthase	3.33
9	A0A4W6FB21111	NADH dehydrogenase (ubiquinone) iron-sulfur protein 5	3.25
10	A0A4W6E7W2	Sodium channel subunit beta-2 isoform X1	3.18
**Decreased abundance in diet B**			
1	A0A4W6DCM7	Guanine nucleotide-binding protein subunit alpha	0.02
2	A0A4W6EGV8	1-phosphatidylinositol 4,5-bisphosphate	0.07
3	A0A4W6DLB9	Cell adhesion molecule 1a	0.08
4	A0A4W6CDB0	Spermine synthase	0.08
5	A0A4W6DXP0	Guanine nucleotide binding protein (G protein)	0.09
6	A0A4W6CNV2	ATP synthase subunit beta (EC 7.1.2.2)	0.09
7	A0A4W6BVV5	Small ribosomal subunit protein uS2	0.09
8	A0A4W6DBS0	ADP ribosylation factor like GTPase 3b	0.10
9	A0A4W6CBE8	Solute carrier family 17 member 6	0.15
10	A0A4W6DBM8	Uncharacterized protein LOC108891021	0.16

* Values > 1.3 indicate more abundant in diet B; values < 0.76 indicate less abundant in diet B.

**Table 7:** Top ten DAPs in the liver tissue.

	Protein ID	Protein Name	Fold Change *
**Increased abundance in diet B**			
1	A0A4W6G873	Pancreatic elastase II	7.13
2	A0A4W6CY37	Chymotrypsin-like	7.04
3	A0A4W6FQC7	Vesicle transport protein USE1	6.99
4	A0A4W6D8E6	Lin-9 DREAM MuvB core complex component	5.05
5	A0A4W6BK05	RNA helicase	4.86
6	A0A4W6DXB6	Dematin actin binding protein	4.74
7	A0A4W6FXS8	Myoglobin	3.91
8	A0A4W6CMS6	O-acyltransferase	3.89
9	A0A4W6F0G8	SPRY domain containing 4	3.81
10	A0A4W6E876	Ring finger protein 7	3.64
**Decreased abundance in diet B**			
11	A0A4W6CQU6	C1q domain-containing protein	0.15
12	A0A4W6FZS4	Zinc finger CCCH-type containing 7B	0.17
13	Q6ITU9	Parvalbumin	0.19
14	A0A4W6EPI8	Ig-like domain-containing protein	0.21
15	A0A4W6BTM5	Uncharacterized protein	0.25
16	A0A4W6BKR3	RNA helicase DDX5	0.25
17	A0A4W6BMF0	CUB domain-containing protein	0.26
18	A0A4W6EUI1	Si: dkey-69o16.5	0.27
19	A0A4W6FRU8	Prothrombin	0.28
20	A8D3J6	Lysozyme g	0.28

* Values > 1.3 indicate more abundant in diet B; values < 0.76 indicate less abundant in diet B.

**Table 8:** Top ten DAPs in the intestine tissue.

	Protein ID	Protein Name	Fold Change *
**Increased abundance in diet B**			
1	A0A4W6DGN9	Threonyl carbamoyl-AMP synthase	9.12
2	A0A4W6CQR5	GIT ArfGAP 1	6.92
3	A0A4W6DG98	NACHT domain-containing protein	5.29
4	A0A4W6EI74	Apolipoprotein Ea	5.16
5	A0A4W6FS56	RUN and SH3 domain containing 1	4.74
6	A0A4W6DP25	YTH domain-containing family protein	4.40
7	A0A4W6D1L2	Metallo endopeptidase	4.20
8	A0A4W6DT98	DnaJ (Hsp40) homolog, subfamily C, member 8	4.03
9	A0A4W6FGH5	Bridging integrator 2b	4.00
10	A0A4W6CAB3	AP complex subunit sigma	3.92
**Decreased abundance in diet B**			
11	A0A4W6DJG2	C1q domain-containing protein	0.06
12	A0A4W6EI75	non-specific serine/threonine protein kinase	0.06
13	A0A4W6BTQ3	UPAR/Ly6 domain-containing protein	0.18
14	A0A4W6FXK4	phosphatidylinositol-4,5-bisphosphate 3-kinase	0.20
15	A0A4W6G0X6	H1.0 linker histone	0.25
16	A0A4W6CTG1	Uncharacterized protein	0.29
17	A0A4W6F148	Zmp:0000000846	0.29
18	A0A4W6BT82	Tyrosine-protein kinase (EC 2.7.10.2)	0.30
19	A0A4W6BTD9	Solute carrier family 25-member 18	0.31
20	A0A4W6ECB4	Fatty acid-binding protein	0.31

* Values > 1.3 indicate more abundant in diet B; values < 0.76 indicate less abundant in diet.

## Data Availability

The mass spectrometry proteomics data have been deposited to the ProteomeXchange Consortium via the jPOST [105] repository with the dataset identifier PXD069810 (https://repository.jpostdb.org/entry/JPST004137, created 24 October 2025). Supplementary Data S1–S9 have been deposited in the Zenodo repository and are publicly accessible via the following: DOI—https://doi.org/10.5281/zenodo.17933445.

## Supplementary Materials

The following supporting information can be downloaded at: https://www.mdpi.com/article/10.3390/proteomes14010006/s1. Supplementary Data S1—Proteins identified in each tissue; Supplementary Data S2—Differentially abundant proteins of brain tissue, Diet A1–4, Diet B1–4; Supplementary Data S3—Functional analysis of differentially abundant proteins of brain using String database; Supplementary Data S4—Differentially abundant proteins of liver tissue, Diet A1–4, Diet B1–4; Supplementary Data S5—Functional analysis of differentially abundant proteins of liver using the String database; Supplementary Data S6—Differentially abundant proteins of intestine tissue, Diet A1–4, Diet B1–4; Supplementary Data S7—Functional analysis of differentially abundant proteins of intestine using the String database; Supplementary Data S8—Isolation list and peptide ratio results obtained from PRM analysis of selected differentially abundant proteins in brain, liver and intestine; Supplementary Data S9—Iron concentration measured by ICP-MS in brain, liver, and intestine.
